# Language impairments in seropositive and seronegative autoimmune encephalitis

**DOI:** 10.1007/s10072-024-07382-2

**Published:** 2024-02-15

**Authors:** Sarah P. Griffith, Robb Wesselingh, Fiore D’Aprano, Nabil Seery, Tiffany Rushen, Chris Kyndt, Brian Long, Udaya Seneviratne, Tomas Kalincik, Katherine Buzzard, Helmut Butzkueven, Terence J. O’Brien, Rubina Alpitsis, Charles B. Malpas, Mastura Monif, on behalf of Australian Autoimmune Encephalitis Consortium

**Affiliations:** 1 Department of Neurosciences, Central Clinical School, Faculty of Medicine, Nursing and Health Sciences, Monash University, Level 6, Alfred Centre, 99 Commercial Road, Melbourne, VIC 3004 Australia; 2 Department of Neurology, Alfred Health, Level 6, Alfred Centre, 99 Commercial Road, Melbourne, VIC 3004 Australia; 3 Melbourne School of Psychological Sciences, The University of Melbourne, Melbourne, VIC Australia; 4 Department of Neurology, Melbourne Health, 300 Grattan Street, Parkville, VIC 3050 Australia; 5 Department of Neurosciences, Eastern Health Clinical School, Monash University, Box Hill Hospital, Melbourne, VIC Australia; 6 Monash Medical Centre, Neuropsychology Unit, Monash Health, 246 Clayton Road, Clayton, VIC 3168 Australia; 7 Department of Neurosciences, Monash Health, Clayton Road, Clayton, VIC 3168 Australia; 8 CORe, Department of Medicine, Royal Melbourne Hospital, The University of Melbourne, Melbourne, VIC Australia

**Keywords:** Autoimmune encephalitis, Language, Cognition, Neuropsychology, Neuroimmunology

## Abstract

**Background and objective:**

Autoimmune encephalitis (AE) is a rare neuroinflammatory disease affecting the central nervous system. To examine language functions in patients with different subsets of AE consisting of seropositive and seronegative groups.

**Methods:**

Fifty-two patients were recruited from neurology departments in Melbourne, Australia, who met clinical criteria for possible AE. Language tests include the Naming Test from the Sydney Language Battery (SydBat), the semantic fluency trial from the Controlled Oral Word Association Test (COWAT), and the Vocabulary and Similarities subtests of the Weschler Abbreviated Scale of Intelligence–Second Edition. The results were standardised with normative data.

**Results:**

The mean age of our cohort was 52.5 years old, with the average time from hospital admission to recruitment being 38.41 months. At an aggregate level, none of the mean language test *z*-scores were below normative data. At the patient level, impairment rates were 18.37% for COWAT (animals), 28.57% for SydBat (naming), 4.65% for Similarities, and 4.55% for Vocabulary. Chi-squared goodness of fit tests indicated that observed performances were significantly below expected performances for the SydBat (naming) test (*p* < 0.0001) and COWAT (animals) (*p* = 0.004).

**Discussion:**

While, on average, language functions were within normal limits in patients with AE, but a subgroup exhibited lower performance in semantic fluency and visual confrontation naming, with impairment rates below expected norms. To advance understanding of language in chronic AE patients, exploring the impact of seizure burden, antiseizure medication use, and the relationship of language functions with other cognitive functions is crucial.

## Introduction

Autoimmune encephalitis (AE) is a rare neuroinflammatory disease that affects the central nervous system [1]. Patients with AE can exhibit a constellation of symptoms, including seizures, behavioural changes, mood disturbance, and cognitive deficits [1].

Specific autoantibodies are linked to distinct constellations of symptoms. For instance, individuals with anti-N**-**methyl-D-aspartate receptor (NDMAR) antibody(ab)-mediated AE typically exhibit psychiatric symptoms, whereas those with anti-leucine-rich glioma-inactivated 1 (LGI-1) ab-mediated AE often present with facial brachial dystonic seizures [1] and/or memory impairment [1]. Patients with autoantibodies in cerebrospinal fluid (CSF) or serum are classified as ‘seropositive’, such as the anti-NMDAR antibody (ab)-mediated or anti-LGI-1 ab-mediated category described above. There is a subset of patients who present clinically with AE symptoms without the presence of currently known antibodies, and these cases are classified and characterised as ‘seronegative’ [1].

Reported clinical language impairments across autoimmune neurological diseases are wide-ranging, and studies apply diverse characterisation of symptomatology, making it difficult to homogenise the language outcomes [2]. These impairments have included ‘aphasia/dysphasia’, ‘verbal fluency problems’, ‘naming difficulties’, ‘word-finding difficulties’, ‘paraphasia’s’, and ‘anomia’ [2]. Psychometrically, a scoping review of language in autoimmune neurological diseases revealed that previous studies on language impairment were often in anti-NMDAR ab-mediated AE patients. These patients often were reported to have language production difficulties, namely in naming and verbal fluency [2]. Naming refers to word finding, typically measured by visual confrontation naming. Verbal fluency refers to the ability to produce spoken language and is typically measured by the quantity of words (either letters or categories) produced in a minute. Few studies had investigated comprehension, the ability to understand, interpret, and make meaning of information; however, outcomes were variable [2]. Overall, the scoping review concluded that many studies across the autoimmune neurological disease did not make use of language tests, or when used, did not report specific tests or specific language deficits.

To assess language, predominantly, tests of visual confrontation naming and semantic retrieval have been used in the AE population. A prospective study examining patients with anti-NMDAR ab-mediated AE revealed impairments in semantic fluency during the acute phase of the illness (50%) and some resolution over time in patients, with 40% of chronic patients demonstrating impairments at least 6 months post-AE [3]. This suggests in some patients that there may be a capacity for recovery of aspects of language functions [3].

In an immunotherapy-naïve cohort of seropositive and seronegative chronic AE patients, the testing of orthographic (e.g. letter) and semantic (e.g. animals) lexical retrieval, as well as visual confrontation naming, yielded results that, on average, fell within age-expected ranges, where impairment was defined at one standard deviation below expectations [4]. However, frequency analysis indicated impairments in 23.5% of the cohort on a naming test, 25.0% on lexical word fluency, and 33.3% on semantic word fluency [4]. A systematic review of anti-NMDAR ab-mediated AE patients in the chronic stage of disease (greater than 12 months after initial administration of immunotherapy) reported language impairments in 28.6% of cases, highlighting that there can be potential persistence of deficits [5]. However, what tests were included in the language measures is unclear. Similarly, another retrospective study of seropositive and seronegative patients identified language impairments in 27.1% of AE patients [6]. In a retrospective case series involving anti-LGI1 ab-mediated AE patients, 20% of the patients exhibited reduced test performance in tests assessing language including reduced semantic fluency and reduced ability in visual confrontation naming, suggesting variable language outcomes [7]. Volumetric analysis using magnetic resource imaging comparing anti-NMDAR ab-mediated AE patients to statistical imaging atlases highlights cortical alterations within the cognitive-linguistic networks [8]. Collectively, although there have been some attempts to characterise language deficits in patients with AE, the picture remains unclear, warranting a broader and in-depth investigation of language in different subtypes of AE.

Given the impact of language impairments on quality of life in other neuroimmunological conditions, such as multiple sclerosis (MS) [9], understanding language function in AE is essential as the ability to communicate and comprehend information is crucial in all aspects of daily life. In other neuroinflammatory diseases, such as MS, it has been demonstrated that language impairment can significantly impact a person’s quality of life [9]. What language deficits occur in different subsets of AE patients and how that impacts the person’s day-to-day functioning is unknown. Understanding language impairment in AE is crucial as it can lead to tailored cognitive rehabilitation programs. This understanding, in turn, facilitates the identification of rehabilitation needs of AE patients.

We aimed to evaluate several language functions in patients who met the criteria for possible AE [1] and were at least 6 months after acute illness. These evaluations included tests of semantic fluency, visual confrontation naming, verbal abstract reasoning, and word meanings.

## Methods

### Participants

Patients were recruited through outpatient neurology clinics at four major metropolitan hospitals in Melbourne, Australia, Alfred Health, Monash Health, Eastern Health, and Melbourne Health as part of the larger Australian Autoimmune Encephalitis Consortium Study. Participation required patients to meet the criteria for possible AE as per Graus and colleagues’ criteria [1] at least 6 months after the diagnosis of AE, with English as their primary language. Those with a diagnosed neurodegenerative disease or currently under investigation for a possible neurodegenerative disease (e.g. dementia of the Alzheimer’s type) were excluded. None of the patients had a history of developmental language disorder or were diagnosed with an intellectual disability. 

### Standard protocol approvals, registrations, and patient consents

The central Human Research Ethics Committee at Alfred Health approved the study (HREC/17/Alfred/168). Patients or the person(s) responsible (in cases where the patient could not consent themselves) provided informed consent.

### Procedure

All patients underwent neuropsychological assessment, including a semi-structured clinical interview conducted by a clinical neuropsychologist. Sociodemographic variables (age, sex, and years of education) and clinical information were collected during the interview. Data regarding immunotherapy treatment were collected, where the first line was classified as IVIg and/or corticosteroids; the second line included rituximab or cyclophosphamide; and the third line included tocilizumab or bortezomib. Data on other clinical and paraclinical variables were collected from medical records when available. They included ICU admission (y/n), mRS (modified Rankin Scale [10]) at discharge, and the number of antiseizure medications (ASMs) at the time of assessment. Due to local COVID-19 restrictions during part of the recruitment phase, five patients were assessed via telehealth. Reasons for incomplete assessments included time limitations (*n* = 5), seizure (*n* = 1), and severely cognitively impaired affecting participation (*n* = *2).*

### Materials

#### Sydney Language Battery—Naming Test

The Naming test of the Sydney Language Battery (SydBat) is a measure of visual confrontation naming. It requires the participant to verbally produce the name of the coloured, photographed item one at a time [11]. The number of correct items is scored out of 30. A *z*-score is derived from the raw score and normative data from healthy controls. Normative data from healthy controls is provided in the manual.

#### Controlled Oral Word Association Test

The category fluency trial of the Controlled Oral Word Association Test (COWAT) was administered. The patients were asked to say as many animals as they can in 1 min. The total score is the number of correct words (animals) produced. Raw scores were converted into *z*-scores, via normative data from Tombaugh and colleagues [12].

#### Wechsler Abbreviated Scale of Intelligence—2nd Edition (WASI-II)

The WASI-II [13] Vocabulary and Similarities subtests were employed. The former measures word meanings, and the latter assesses verbal abstract reasoning. Normative data is provided in the manual, with raw scores converted to *T*-scores. *T*-scores were then converted to *z*-scores.

### Statistical analysis

Psychometric ‘impairment’ was defined as a score falling 1.5 standard deviations (SD) or more below the normative mean, as this is sensitive enough to detect cognitive dysfunction while maximising specificity ^14^.

Summary statistics were derived for cohort demographics and neuropsychological measures, with analysis conducted on JASP (version 0.16.3)15. Graphs were created using GraphPad Prism (version 9.0.0) and Microsoft Excel (Version 16.66.1). Missing data were treated with pairwise deletion.

Test means were subject to correlation analyses to examine their relationship to demographic variables and are reported as Spearman’s rank correlation coefficients. The variable ‘time between symptom onset to recruitment’ demonstrated significance in this correlation analysis. Subsequently, we estimated a regression model to predict test scores based on this identified time interval, with further adjustments made for age within the model. An outlier on the variable ‘time between symptom onset to recruitment’ was identified during data visualisation. Upon closer examination, this outlier was found to be a patient with anti-NMDAR positivity who exhibited symptoms before the identification of the anti-NMDAR antibody, which was first reported in 2008. Regression models were executed both with and without the inclusion of the outlier due to the exploratory nature of this paper.

The test means of the seropositive and seronegative groups were subject to an independent *t*-test to assess for significant differences. Non-parametric tests (Mann-Whitey *U* test) were applied across the tests for uniformity because some of the parametric assumptions were violated. Statistical assumptions were checked using Levene’s test (homogeneity variance) and the Shapiro–Wilk test (normality).

A comparison of the frequency of observed outcomes to expected outcomes was performed employing chi-squared goodness of fit tests. The expected outcome was defined as psychometric impairment, which was classified at 1.5 standard deviations below the normative mean, where 6.7% of the patients in a normative cohort would be classified as impaired.

Psychometric patterns were ascertained through pattern analysis. Pattern analysis involves the computation of the number of distinct patterns of psychometric findings. In pattern analysis, missing data were managed through list-wise deletion.

## Results

Fifty-two patients met inclusion criteria, of which 27 (52%) were female, 26 (50%) were seropositive; 11 (42.31%) with anti-NMDAR ab-mediated AE, 10 (38.46%) with anti-LGI-1 ab-mediated AE, 2 (7.69%) with contactin-associated protein-like 2 (CASPR-2) ab-mediated AE, and 1 (3.85%) with voltage-gated potassium channel complex (unspecified; VGKC) ab-mediated AE antibodies, and 2 (7.69%) with another antibody. Demographic data are available in Table 1.

**Table 1:** Total cohort demographics

Characteristics		Mean (SD) [range]	*N*
Age, years		52.5 (18.32) [21–81]	52
Sex, female (*N* (%))		27 (51.92)	52
Months between symptom onset and neuropsychological assessment		38.39 (32.13) [6–126]	51
Months between hospital admission and neuropsychological assessment		38.41 (31.39) [6–121]	51
Months between symptom onset to hospital admission		2.60 (5.42) [0–30]	51
Education, years		12.82 (2.84) [7-18]	52
Telehealth, *y* (*N* (%))		5 (9.61)	52
Seropositive, *y* (*N* (%))		26 (50)	52
	Anti NMDAR ab-mediated AE	11 (42.31)	26
	Anti LGI-1 ab-mediated AE	10 (38.46)	26
	Anti CASPR2 ab-mediated AE	2 (7.69)	26
	Anti VGKC ab-mediated AE	1 (3.85)	26
	Other antibodies	2 (7.69)	26
Treatment line, *y* (*N*%))	First line	51 (100)	51
	Second line	27 (52.941)	51
	Third line	0 (0)	51
ASM use, *y* (*N* (%))	0 ASM	25 (50.00)	50
	1 ASM	12 (24.00)	50
	2 + ASM	13 (26.00)	50
ICU admission during main hospital admission, *y* (*N* (%))		22 (47.83)	46
mRS at discharge (*N* (%))	1	9 (19.56)	46
	2	18 (39.13)	46
	3	16 (34.78)	46
	4	3 (6.52)	46

*Ab* antibody, *NMDAR* N-methyl-D-aspartate receptor, *LGI-1* anti-leucine-rich glioma-inactivated-1, *CASPR2* contactin-associated protein-like 2, *VGKC* voltage-gated potassium channel complex, *ASM* anti-seizure medication, *ICU* intensive care unit, *mRS* modified Rankin Scale

**Fig. 1:**
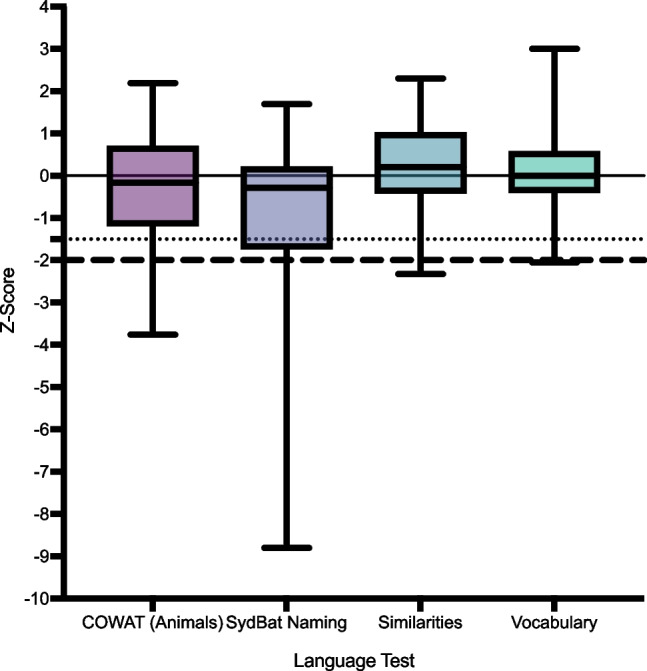
Scores of language tests presented as box plots. Normative data mean is denoted by a black line. Scores below the dotted lines are 1.5 standard deviations below the normative mean and are considered mildly impaired. Scores below the dashed line are − 2.0 standard deviations below the normative mean and are considered severely impaired

Four patients (7.69%) spoke English as an additional language. The average length of English as their primary language was 40.25 years (range 10–56).Language test characteristics are avaliable in Table 2, with visualisation in Fig. 1.

**Table 2:** Subtests characteristics

	Cohort *N*	*M* (SD)	Min	Max	*N* (%) impaired at − 1.5 SD below mean
*Total cohort*					
COWAT (animals)	51	− 0.24 (1.39)	− 3.76	2.19	9 (18.37)
SydBat (naming)	51	− 0.88 (1.99)	− 8.80	1.69	15 (29.41)
WASI-II Similarities	44	0.20 (0.98)	− 2.33	2.30	2 (4.65)
WASI-II Vocabulary	45	0.11 (0.93)	− 2.05	3.00	2 (4.55)
*Seropositive*					
COWAT (animals)	26	− 0.28 (1.26)	− 2.57	2.19	4 (16.67)
SydBat (naming)	26	− 0.77 (1.59)	− 5.50	1.14	8 (33.33)
WASI-II Similarities	21	0.21 (0.82)	− 1.00	1.90	0 (0.00)
WASI-II Vocabulary	21	0.18 (0.82)	− 1.30	2.80	0 (0.00)
*Seronegative*					
COWAT (animals)	25	− 0.32 (1.52)	− 3.76	2.00	5 (20.00)
SydBat (naming)	25	− 0.95 (2.41)	− 8.80	1.69	7 (28.00)
WASI-II Similarities	23	0.23 (1.13)	− 2.33	2.30	2 (8.70)
WASI-II Vocabulary	24	0.05 (1.04)	− 2.05	3.00	2 (8.33)

*COWAT* Controlled Oral Word Association Test, *SydBat* Sydney Language Battery, *WASI* Wechsler Abbreviated Scale of Intelligence

## Language test characteristics

## Correlations with demographic data

When assessing for verbal fluency, greater time between symptom onset and cognitive assessment (recruitment) was associated with lower COWAT (animals) *z*-scores (*r*(48) = − 0.32,* p* = 0.03). Greater time between hospital admission and recruitment was associated with lower scores on COWAT (animals) *z*-scores (*r*(48) = − 0.30,* p* = 0.03). Lower scores on the SydBat (naming) test was associated with higher number of ASM (0, 1, 2 +) (*r*(47) = − 0.35,* p* = 0.02). Higher scores on the SydBat (naming) was associated with greater years of education (*r*(48) = 0.33* p* = 0.02). Higher scores on Similarities (WASI-II) and higher scores on Vocabulary (WASI-II) were both associated with greater years of education, (*r*(42) = 0.45,* p* = 0.003) and (*r*(43) = 0.55,* p* < 0.001), respectively..

Given the significant relationship between the findings involving longer time intervals and lower COWAT scores, a regression model was estimated to predict test means based on the time between symptom onset and recruitment, adjusting for age. On the initial simple linear regression model, the time between symptom onset and recruitment was not associated with scores on the COWAT (animals) ($\beta$ = − 0.22, *p* = 0.13).

Visual inspection of the time between symptom onset and recruitment data revealed an outlier, with 220 months between symptom onset and recruitment. Upon closer examination, this outlier was found to be a patient with anti-NMDAR positivity who exhibited symptoms before the identification of the anti-NMDAR antibody, which was first scientifically reported in 2008 [14]. When this outlier was removed from the linear regression, longer time between symptom onset and recruitment was associated with lower scores on the COWAT (animals), ($\beta$ = − 0.34, *p* = 0.02). This association persisted when adjusting for age ($\beta$= − 0.32, *p* = 0.03). Without this outlier, the time between symptom onset and recruitment was not associated with SydBat (naming) *z*-scores ($\beta$ = − 0.13, *p* = 0.37), Similarities ($\beta$ = − 0.15, *p* = 0.36), and Vocabulary ($\beta$ = 0.00, *p* = 0.97).

## Independent sample *t*-tests

Table 3 presents results for the independent sample *t*-test comparing serostatus groups.

**Table 3:** Independent *t*-test comparing cases of seropositive AE versus seronegative cohort serostatus

	Group	*N*	Mean	SD	*W*	*p*
COWAT (animals)	Seronegative	25	− 0.318	1.521	322.00	0.67
	Seropositive	24	− 0.280	1.264		
SydBat (naming)	Seronegative	25	− 0.945	2.414	310.50	0.84
	Seropositive	24	− 0.774	1.591		
WASI-II Similarities	Seronegative	23	0.229	1.133	245.00	0.72
	Seropositive	20	0.207	0.817		
WASI-II Vocabulary	Seronegative	24	0.050	1.039	223.00	0.70
	Seropositive	20	0.179	0.816		

## Frequency analysis

### Total cohort

Nine (17.65%; 95% CI [8.4, 30.87]) patients were impaired on COWAT (animals). Fifteen (29.41 95% CI [17.49, 43.83]) patients were impaired on the SydBat (naming). Two patients were impaired on the similarities and vocabulary tests (4.55% 95% CI [0.56–15.47]; 4.44% 95% CI [0.54, 15, 15]), respectively.

Chi-squared goodness of fit tests were conducted to compare the actual number of impaired patients to the expected number of impaired patients under a normal distribution, where impaired is defined at 1.5 standard deviations below the normative mean. This revealed significant differences for the SydBat (naming) (*χ*^2^(1, *N* = 51) = 51, *p* < 0.00001), with the cohort performing poorer than expected. There was a significant difference for the COWAT (animals) (*χ*^2^(1, *N* = 51) = 12.7, *p* = 0.004), with the cohort performing poorer than expectations. There was not a significant difference between observed and expected performances for Similarities (*χ*^2^(1, *N* = 44) = 0.36,* p* = 36) and Vocabulary (*χ*^2^(1, *N* = 45) = 0.36,* p* = 0.55).

### Seropositive AE cohort

Four (15.38%; 95% CI [4.36, 34.87]) patients are impaired on COWAT (animals). Nine (34.62 95% CI [17.21, 55.67] patients are impaired on SydBat (naming). None of the seropositive patients were impaired on the similarities or vocabulary tests (0.00%; 95% CI [0.00, 16.11], 0.00% 95% CI [0.00, 16.84]).

Chi-squared goodness of fit tests were conducted for the seropositive group. This revealed significant differences for the SydBat (naming) (*χ*^2^(1, *N* = 26) = 26.54,* p* < 0.00001), with the cohort performing poorer than expectations. These tests did not reveal significant differences between observed and expected performances for COWAT (*χ*^2^(1, *N* = 26) = 2.16,* p* = 0.14), Similarities (*χ*^2^(1, *N* = 21) = 1.05,* p* = 0.31), and Vocabulary (*χ*^2^(1, *N* = 20) = 1.05,* p* = 0.31).

### Seronegative AE cohort

Five patients were impaired on COWAT (animals) (20.00%, 95 CI [6.83, 40.70]). Six patients were impaired on SydBat (naming) (24.00%; 95 CI [9.36, 45.13]), while 2 patients were impaired on Similarities and Vocabulary, 8.70%; 95 CI [1.07, 28.04] and 8.33%; 95 CI [1.03, 27.00], respectively.

Chi-squared goodness of fit tests were conducted for the seronegative group. This revealed significant differences for the COWAT (animals) (*χ*^2^(1, *N* = 25) = 4.89,* p* = 0.03) and SydBat (naming) (*χ*^2^(1, *N* = 25) = 8.69,* p* = 0.003), with both suggesting the cohort performed poorer than expectations. These tests did not reveal significant differences between observed and expected performance for the Similarities (*χ*^2^(1, *N* = 23) = 0,* p* = 1) and Vocabulary (*χ*^2^(1, *N* = 24) = 0,* p* = 1).

## Pattern analysis

Using pattern analysis, of the 16 possible patterns of language outcomes to emerge from the data, 7 were observed (Table 4). The most common emerging pattern was ‘normal language’ psychometrics (62.80%). The second most frequent was SydBat (Naming) test impairment (18.60%).

**Table 4:** Pattern of language outcomes

Pattern of psychometric impairment	*N*	%
Normal language psychometrics	27	62.80
SydBat (naming) impaired	8	18.60
COWAT (animals) impaired	3	6.98
COWAT (animals) and SydBat (naming) impaired	2	4.65
Similarities subtest impaired	1	2.33
Vocabulary subtest impaired	1	2.33
Global impairments	1	2.33
Total	43	

## Discussion

We investigated language function in a cohort of AE patients during the chronic stages of the disease. These tests focused on semantic fluency, visual confrontation naming, and vocabulary knowledge. Notably, in comparison to normative data, our overall results revealed that, on average, language functions, when evaluated in the entire AE cohort or the seronegative and seropositive subgroups, exhibited no significant impairments. However, a detailed frequency analysis indicated impairments in a subgroup of patients. Specifically, performance on semantic fluency and visual confrontation naming tests was impaired in approximately one-fifth of the total cohort, both in seropositive and seronegative subgroups. Conversely, verbal abstract reasoning and word meaning tests were less frequently impaired. Although patients or their carer may report symptoms indicative of possible language deficits, on comprehensive testing, our results demonstrated that a large proportion of AE patients had intact language.

Tests of abstract verbal reasoning and word meanings had lower rates of impairment compared to confrontation naming and semantic fluency tests. Further, the rates of abnormal test scores on verbal abstract reasoning and word meanings were consistent with those expected in a normative cohort of healthy control participants; that is, it was consistent with expected rates of impairment in the general population. These tests are often considered markers of hold functions—namely, these tests are relatively stable over time and relatively insensitive to cognitive deterioration [15]. Together, these findings may suggest that these tests might not be as sensitive to the subtle changes in language function in chronic AE cases.

In contrast, a subset of patients, regardless of serostatus, demonstrated psychometric impairment on tests of visual confrontation naming (approximately 30% of the sample) and semantic fluency (approximately 20% of the sample). Further, when examining expected versus actual impairment rates, the total cohort performed poorer than expectations on visual confrontation naming and semantic fluency. When considered by seropositive and seronegative groups, both groups had poorer performance on visual confrontation naming, while only the seronegative group was poorer than expectations on the semantic fluency test. Of interest, these results were consistent with a recent study of patients with MS, another neuroinflammatory disease of the CNS [16]. The study also employed normative data and reported approximately a quarter of patients demonstrated impairment in the Boston Naming Test (BNT), a visual confrontation naming test. Similarly, approximately one-fifth of their sample was impaired on a semantic fluency test. Further investigation into other neuroinflammatory conditions is needed to ascertain the potential involvement of neuroinflammatory markers in these outcomes.

The observed impairments to language in AE are plausibly underpinned by secondary factors that undermine or produce a vulnerability within broadly distributed language networks. When considering cognitive factors, confrontation naming and semantic fluency entail several stages or processes. In the former, the initial stage involves perceptual identification of the image [17]. This triggers the activation of the semantic representation, which, in turn, leads to the retrieval of the name, accessing the phonological representation associated with the semantic concept [17]. Ultimately, the correct naming is achieved through the motor programming stage. In semantic fluency, individuals search for and generate words by using clustering, often through semantic categorisation [19]. This process activates functions in the temporal lobe related to verbal memory and word retrieval [19]. Additionally, there is a need to switch and establish new semantic subcategories, which requires the engagement of frontal lobe processes, including strategic search and cognitive flexibility [19].

Consequently, both confrontation naming and semantic fluency tests rely on semantic access [17, 19]. This reliance is further evidenced in patients with neuronal degradation (e.g. Alzheimer’s disease) or neuronal disruption (e.g. temporal lobe epilepsy) to regions that support semantic access often have poorer performances on these tests compared to healthy controls. Theoretically, it is proposed that these tests have differing levels of access to lexical-semantic memory, whereby confrontation naming is automatic, while semantic fluency requires deliberate and effortful access [20]. Given the tests’ reliance on semantic access, the results of these tests might represent a reduction in semantic processing in some patients following AE.

Further, working memory has been implicated in verbal fluency [21] and confrontation naming [18], whereby confrontation naming requires temporarily storing and manipulating information, such as aspects of the image. Semantic fluency requires temporarily storing and manipulating retrieved words, tracking already given words, and inhibiting incorrect works, all while maintaining a search strategy. Consequently, executive dysfunction may also play a role in reduced visual confrontation naming and semantic fluency in this cohort. Additionally, it is important to consider the potential impact on fundamental cognitive functions, such as primary attention and processing speed, as a decrease in these functions hinders performance on both tests.

Beyond primary neuronal damage, the observed impairments to language in AE are plausibly underpinned by secondary factors that undermine or produce a vulnerability within broadly distributed language networks. These relate in part to the role of seizure activity, and ultimately seizure frequency, in disrupting key cognitive-linguistic functions, as well as the role of ASM usage [22]. Notably, performance on these tests was not significantly different between the serostatus groups, and frequency rates of impairments across seropositive and seronegative patients are similar. Of interest, the number of ASMs used was positively correlated with impairments in visual confrontation naming. Although we did not collect data on this at the time of assessment, we can postulate that patients taking ASMs have been previously diagnosed with epilepsy or have recently experienced seizure activity necessitating ASM treatment. The pancerebral impact of seizure burden and ASM on network dysfunction may promote the inefficiency observed in visual confrontation naming [23].

Of particular interest was the observation of a decline in semantic fluency over time since the initial onset of symptoms. This trend persisted even after accounting for age as a potential factor. Notably, no such temporal relationship was discerned in the case of the other language tests administered. Several factors may contribute to these findings. Firstly, since semantic fluency is the sole-timed test within the battery of language tests, this decline could be attributed to a reduction in processing speed [19]. Such slowed processing speed might, in turn, be a consequence of ongoing drug-resistant seizures [23–25]. Secondly, it is plausible that these findings reflect a recruitment bias. This bias could be due to the fact that our patient cohort was recruited from neurology clinics. It is conceivable that those patients who continue to receive long-term clinical follow-up may exhibit more pronounced cognitive deficits, functional limitations, and perhaps even more intractable seizure outcomes. As such, these findings may partially mirror the influence of this recruitment bias. Replication of these findings in a larger sample size is important to confirm the veracity of these findings.

In a broader examination of the ramifications of AE-associated seizures on language, it becomes imperative to consider the wider spectrum of language impairments associated with seizure disorders, such as epilepsy. Notably, individuals with epilepsy frequently exhibit language issues, an outcome well-documented in the context of dominant hemisphere temporal lobe epilepsy [26]. Existing studies often focus on visual confrontation naming tests, such as the Boston Naming Test (BNT), where significant impairments have been observed in patients compared to controls [27]. However, recent investigations have drawn attention to more nuanced and subtle language effects in seizure disorders affecting aspects of high-level language beyond the single word level [28, 29], prompting a need for a similarly nuanced consideration within the study of language impairments following AE [27].

It is crucial to acknowledge that a direct comparison between these two populations presents inherent challenges. Many studies on language impairments in epilepsy involve individuals with longstanding seizure disorders, spanning several years. Contrastingly, the duration of seizures in those with AE varies widely, making it challenging to draw direct parallels. Additionally, the absence of clear epileptic zone localisation in many patients with AE further complicates the comparison. Determining whether language effects stem from localised damage or pan-cerebral impact of seizure burden, coincidentally involving language alongside other cognitive domains, remains elusive. Consequently, a more in-depth exploration of the interplay between seizures and cognition in the context of AE is warranted.

Significant correlations were observed between education and performance on verbal abstract reasoning, word meanings, and visual confrontation naming tests. This finding aligns with expectations, as previous research has established that education influences visual confrontation naming scores across various groups [30]. Moreover, when examined within the framework of the Cattell-Horn-Carroll theory, both the verbal abstract reasoning and word meaning tests are heavily weighted toward crystallised knowledge [31]. As a result, they are more likely to be resilient to the impact of brain injuries, making the connection between education and these language measures unsurprising.

Several limitations should be acknowledged. First, this study did not assess other language functions, such as repetition or comprehension, potentially limiting the comprehensive examination of language abilities. Additionally, a qualitative analysis of day-to-day language was not conducted, which could have provided deeper insights into the data, particularly in relation to high-order language functions. Another noteworthy limitation is the absence of corrections for multiple comparisons due to the relatively small sample sizes. While this increases the risk of false positives and the potential for obtaining statistically significant results by chance, we have accepted this higher rate as this is an exploratory study of a rare disease. Furthermore, detailed information regarding seizure frequency and specific types of epilepsy was not obtained, which could have added valuable context to the findings. In addition, we did not have MRI data to discern the extent of visible neuroinflammation or subregional changes and their correlation to these outcomes. Lastly, the visual confrontation naming test presented certain limitations; the visual confrontation naming normative data exhibits a notable skew toward individuals of older age [11] and higher education levels. This poses two limitations; first, the generalisability of the results may not be applicable given these demographics. Second, a number of our participants were below this demographic age range. If we consider that visual confrontation naming declines with older age [30], this raises the possibility that individuals within our cohort of a younger age who manifest difficulties in confrontation naming may have more pronounced cognitive impairments than the current results suggest.

## Conclusion

We evaluated language functions in 52 patients with AE at least 6 months after initial illness. While language functions were intact on average, a sub-group of patients demonstrated reduced semantic fluency and visual confrontation naming performance. The rate of observed impairments in these functions was significantly below the expected rates based on normative data. To advance the understanding of language in chronic AE patients, exploring the impact of seizure burden, antiseizure medication use, and the relationship of language functions with other cognitive functions in individuals with chronic AE is crucial.

## Data Availability

The datasets generated during and/or analysed during the current study are available from the corresponding author on reasonable request.
